# Correction: Monitoring progress towards elimination of hepatitis B and C in the EU/EEA

**DOI:** 10.1371/journal.pgph.0001907

**Published:** 2023-05-02

**Authors:** Katherine C. Sharrock, Teymur Noori, Maria Axelsson, Maria Buti, Asuncion Diaz, Olga Fursa, Greet Hendrickx, Cary James, Irena Klavs, Marko Korenjak, Mojca Maticic, Antons Mozalevskis, Lars Peters, Rafaela Rigoni, Magdalena Rosinska, Kristi Ruutel, Eberhard Schatz, Thomas Seyler, Irene Veldhuijzen, Erika Duffell

In the Data Availability Statement, an incorrect link is provided. The Data Availability Statement should read: All data presented is available in an online report available at https://www.ecdc.europa.eu/sites/default/files/documents/hepatitis-B-C-monitoring-responses-hepatitis-B-C-epidemics-EU-EEA-Member-States-2019_0.pdf.
