# Supplementary material for: Monitoring progress towards elimination of hepatitis B and C in the EU/EEA
Source: PLOS Glob Public Health. 2022 Aug 17;2(8):e0000841. doi: 10.1371/journal.pgph.0000841 (PMC10022013; doi:10.1371/journal.pgph.0000841)
Supplement: S1 Table — (DOCX) [file pgph.0000841.s001.docx]

**Supplementary material**

**S1 Table**

Details of the existing data sources included in the monitoring system [6]

| **Data source** | **Indicator** | **Details** |
| --- | --- | --- |
| ECDC | - Prevalence of hepatitis B and C in the general population and risk groups - Infection prevention and control staff - Incidence of acute HBV infection notifications - Hepatitis testing policy - Vaccination of healthcare workers - HBV vaccination policy | [ECDC Hepatitis B Prevalence Database](https://ecdc.europa.eu/en/all-topics-z/hepatitis-b/tools/hepatitis-b-prevalence-database)  [ECDC Hepatitis C Prevalence Database](https://ecdc.europa.eu/en/all-topics-zhepatitis-ctools/hepatitis-c-prevalence-database)  [Point Prevalence survey of healthcare associated infections and antimicrobial use in European acute care hospitals](https://ecdc.europa.eu/sites/portal/files/media/en/publications/Publications/healthcare-associated-infections-antimicrobial-use-PPS.pdf)    [ECDC Surveillance Atlas](https://atlas.ecdc.europa.eu/public/index.aspx)  [ECDC Technical report Hepatitis B and C testing activities, needs, and priorities in the EU/EEA](https://ecdc.europa.eu/sites/portal/files/documents/HepatitisBC-testing-in-EU-May2017.pdf)  ECDC survey of strategies used in EU/EEA Member States for protection of hospital-based healthcare workers and third parties (ECDC, Technical report pending publication) [10]  [ECDC vaccine scheduler](https://vaccine-schedule.ecdc.europa.eu/) |
| EMCDDA | - Estimates of the size of the PWID population - Syringes distributed - Number of people receiving OST - National hepatitis policy inclusive of PWID - Testing policies in harm reduction services and prisons | [EMCDDA Statistical Bulletin](http://www.emcdda.europa.eu/data/stats2019_en)  [EMCDDA Barometer](http://www.emcdda.europa.eu/activities/promoting-hcv-hepatitis-c-virus-testing-and-linkage-care-drugs-services#section2) |
| WHO/UNICEF Joint Reporting Process | - Coverage of third dose of hepatitis B vaccine (Policy and coverage) - National provision of a birth dose of HBV vaccine (Policy and coverage) | [WHO immunization, Vaccines and Biologicals – Data, statistics and graphics](https://www.who.int/immunization/monitoring_surveillance/data/en/) |
| WHO/Europe survey | - HBV vaccination for key risk groups (policy and coverage for healthcare workers) - HAV vaccination for general population and key risk groups (policy) - National antenatal HBV screening programme (policy and coverage) - Provision national antenatal screening programme HCV - Post-exposure prophylaxis of children born to mothers with HBV (policy and coverage) - Provision of antiviral treatment for pregnant women with HBV | WHO/Europe survey on hepatitis immunization and prevention of perinatal transmission – data collected from European countries during 2019 but currently unpublished |
| WHO Health in Prisons European Database (HIPED) | - HBV vaccination availability in prisons - HBV vaccination coverage in prisons | [WHO Health in Prisons European Database](https://apps.who.int/gho/data/node.prisons) |
| EUROSTAT | - Deaths from hepatocellular carcinoma, cirrhosis and chronic liver diseases - Estimates of migrant populations | <https://ec.europa.eu/eurostat/data/database> |
| Council of Europe | - Source of blood donations - HBV and HCV infections among blood donors - HBV and HCV infections among blood donor recipients | [Report on the collection, testing and use of blood and blood components in Europe](https://www.edqm.eu/en/blood-transfusion-reports-70.html) |
| European Men-Who-Have-Sex-With-Men Internet Survey (EMIS-2017) | - Condom use in MSM - Vaccination against HAV and HBV among MSM - Prevalence of HBV or HCV among HIV infected MSM | [European men who have sex with men internet survey](http://www.emis2017.eu) |

*Abbreviations: ECDC – European Centre for Disease Prevention and Control; EMCDDA – European Monitoring Centre for Drugs and Drug Addiction; HAV – hepatitis A virus; HBV- hepatitis B virus; HCV – hepatitis C virus; MSM – Men who have sex with men; OST – Opioid substitution therapy; PWID – person who injects drugs; SDG – sustainable development goals.*
