# Supplementary material for: Monitoring progress towards elimination of hepatitis B and C in the EU/EEA
Source: PLOS Glob Public Health. 2022 Aug 17;2(8):e0000841. doi: 10.1371/journal.pgph.0000841 (PMC10022013; doi:10.1371/journal.pgph.0000841)
Supplement: Attachment — Submitted filename: Response to comments received from reviewers.docx [file pgph.0000841.s002.docx]

Response to comments from Reviewer #1:

**Major comments:
1. It is highly concerning that the sources of data were lacking for many countries. The authors fully own this as a limitation, but it calls into question the validity of the reported results. Can the authors provide examples of the “modelling or surveillance data”? Could these sources be confirmed in any way? Can the data be replicated? This needs to be addressed prior to publication.**

The lack of data is a serious limitation and one of the aims of the paper is to highlight this in order to identify the areas that need addressing.

Examples have been added for modelling and surveillance data.

The point about whether the data can be replicated is important and this is why ECDC is developing a sentinel system. We have added a sentence into the discussion to note this.

**2. Authors should provide context for global hepatitis B and C elimination efforts beyond the EU and EEA. What percentage of the 31 countries are considered high-income? What are the implications for low-income countries if high-income countries are falling short of reporting and elimination goals?**

This is an excellent point and a sentence has been added to the discussion to highlight the wider implications of this data collection.

**3. Why did authors choose to report based on 2020 rather than 2030 SDG goals? The report is outdated (reporting 2017 data in 2022) and I believe readers would be more interested in looking forward rather than backwards. The authors reference both 2020 and 2030 goals, making it unclear which targets are being evaluated here.**

The year 2020 is chose as this is the year of the interim targets for the European Action Plan. This has been made more clear in the introduction.

**4. Can authors comment on whether the chosen indicators should be shifted? Perhaps the criteria for the goals are too stringent such that the goals will not be achievable? For instance, Polaris Observatory Collaborators recommend a shift to using absolute targets as a better measure of true elimination (**[**https://onlinelibrary.wiley.com/doi/abs/10.1111/jvh.13412**](https://onlinelibrary.wiley.com/doi/abs/10.1111/jvh.13412)**).**

WHO have shifted the elimination targets to include absolute targets for impact – incidence and mortality. However, we did not prioritise these as the core indicators and we have chosen to not focus on these here but these will be presented in a future paper. For this reason and in the interest of the word count we have not revised the paper to focus on these changes.

**5. Regarding infant vaccination, the authors should report the vaccination schedules for the various countries, whether in the main file or in a supplementary file. It is unclear as written what the different schedules are and which countries include a birth dose of HBV vaccine. This makes the measurements shown in Table 2 difficult to interpret. It is striking that Denmark, Finland and Iceland do not have national policies. Perhaps the authors should recommend universal vaccination in the Discussion?**

Table 2 has been revised to include the hepatitis B vaccination schedule to provide greater clarity as suggested.

The discussion has been revised with regards to the suggestion around recommending vaccination.

**6. Consider restructuring the discussion section to focus separately on HBV and HCV rather than combining.**
Thank you for this comment. We chose to keep the structure focused on the key building blocks of the monitoring system from prevention through to treatment.

**Minor comments:
1. Introduction – goal 3, “to promote health and wellbeing”, should be placed in quotations.**

The quotation marks have been added.

**2. Introduction – last sentence of 1st paragraph – remove “in 2015” since you referenced 2017 at the beginning of the sentence. Otherwise, make it clear to what “in 2015” refers.**

The “in 2015” has been deleted.

**3. Introduction – paragraph 3, insert a comma before “in alignment with the SDGs”.**

Text has been revised.

**4. Materials and Methods – paragraph 1, insert a comma after “in consultation with an advisory group”**

Text has been revised.

**5. Results – Completeness and data sources section – 2nd sentence of 2nd paragraph – what is meant by “older treatment data”?**

Revision of this sentence to clarify and correct a typo.

**6. Results – Policy section – Start a new sentence beginning, “Of the 31 countries…”**

Text has been revised.

**7. Results – Continuum of care section – Chronic HBV and HCV cases diagnosed – 2nd and 3rd** **paragraphs - Recommend moving the list of the 4 and 7 countries as follows: “four (Denmark, Ireland, Netherlands, UK (Scotland)) of the 12 countries…”**

Text has been revised.

**8. Results – Continuum of care section – Recommend changing the title of subsection ii to “Eligible cases treated”**

Text has been revised.

**9. Discussion – paragraph 2 – please provide a citation and/or overview for the “data…previously collected by WHO Europe” regarding vertical HBV transmission.**

Text revised to clarify the process. There is a citation for the results of the data.

**10. Discussion – paragraph 4 – 2nd sentence – a word seems to be missing after “next monitoring”. Perhaps “next monitoring period”? When is this planned?**

Text has been revised.

**11. Discussion – paragraph 5 – 7th sentence – remove the comma after “healthcare settings”**

Text has been revised.

**12. Discussion – paragraph 6 – what is meant by “sampling for exampling”?**

This is a typo. Text has been revised.

**13. Discussion – paragraph 8 – recommend rewording the 1st sentence for clarity’s sake**

Text has been revised for clarity.

**14. Discussion – paragraph 8 – authors should provide a citation if making a claim such as “it is likely that the majority of those on treatment are virally suppressed”**

A reference has been added.

**15. Discussion – paragraph 12 – 1st sentence - remove the comma after “carried out in 2021”**

Text has been revised.

**16. Discussion – final paragraph – 4th sentence – add a comma between “monitoring systems” and “ensuring adequate resources…”**

Text has been revised.

**17. General – Please revise the following run-on sentences and/or sentences with unclear wording:
o Materials and Methods – 1st sentence**

Text reworded for clarity.

**o Results – Completeness and data sources section – 1st sentence of 2nd paragraph**

Text reworded for clarity.

**o Discussion – 1st sentence of 5th paragraph;**

Text reworded for clarity.

**4th and 7th sentences of 6th paragraph**

Text reworded for clarity.

**Reviewer #2: The manuscript by Sharrock, et al. attempts to quantify progress toward HBV and HCV elimination in the European region. The data is already five years old limiting its utility and the most robust portion of the study appears to be data from other reports (e.g., WHO/UNICEF, EMCDDA...), which are already publicly available. The data collected by ECDC has major gaps including well known available data that are collected/reported by countries as part of their national registry. No attempt was made to collect national registry data but instead the preferred methodology was to send a questionnaire to the countries to fill out on their own.**

The data were collected from countries through the formal routes of nominated national contact points for ECDC. In most instances these are public health institutes or ministries of health and these are the institutions best placed to have a clear over view of the national data. Some of the data does indeed come from registries. However, ECDC did not go searching for local clinical registries but instead operated through these formal contact points. This work has obviously exposed many gaps and ECDC, as noted in the document, is now working with national contact points to explore the use of validated alternative data. In some instances this may indeed come from local registries.

**When limited data is available, researchers are faced with two options – not publish the data because they do not meet a minimum quality threshold, or, publish data and state the limitations. The latter option is influenced by a key decision – does our newly reported data add to the knowledge of the field? The authors believe that the reported data, despite numerous limitations, expand our knowledge with regards to HBV and HCV elimination.**

The data are indeed of low quality. This is a major challenge for hepatitis. We have been explicit throughout the paper in relation to the limitations of the data and have been careful in the conclusions drawn. We still believe that it is important to publish this work in order to highlight these gaps and to draw attention to the work that needs to be done to address data quality.

**There are some major gaps in the study. HBV birth dose vaccination rates that are part of every WHO guideline are missing and only discussed briefly in the discussion section. In addition, total infections for HCV and HBV are missing as well as the references for the original sources. The authors state that national representatives provided this data but what were the sources and the quality of this data. Finally, there are an obvious mythology issue. The percent treated of total diagnosed favors countries with low diagnosis rate. Thus, for HCV, Romania and Bulgaria rank higher than France and UK which have had well established national elimination programs. For HBV, the authors state the complexity of assessing progress for HBV due to treatment eligibility criteria. It would have been very helpful if they had collected and reported country specific HBV treatment eligibility criteria. That would have expanded our knowledge of the field.**

This paper is not intended to present all the data collected but highlight the progress towards the core indicators. These did not include birth dose vaccination as only five countries in the EU have this policy. Additional detail regarding the sources of the data is available in the full report written by ECDC which is clearly noted now in the paper and referenced. Full information on all the data collected including the estimates of the numbers diagnosed are provided in full in the ECDC report that is clearly referenced.

The text has been revised with regards to the issue over the treatment indicator. Information on treatment eligibility was not collected as part of this survey but we are rolling out a sentinel system in clinical sites (which is noted in the paper) and this data will be collected.

**Please allow me to disagree with the authors decision to submit this work for publication. The data is old/outdated, there are major gaps in the data, and insufficient references to original data have been provides. This study does not expand our knowledge but in fact leads to confusion.**

We disagree with this proposal. The data represent the first collection of data from the region that set the baseline for monitoring progress towards the elimination targets. The EU/EEA is the only region globally that has been able to undertaken such a comprehensive data collection with a systematic approach and validation of the data. The data provided is original data provided through formal national public health authorities as noted in the methods. Whilst the data are now indeed a few years old the publication of this information is important in highlighting the challenges of monitoring and the gaps in local data collection systems and responses. By not acknowledging these challenges the problem will not be exposed and will remain unaddressed.

**Detailed comments:**

**• Page 3, paragraph 2 – It is stated that “Elimination is defined as a 65% reduction in hepatitis-related deaths and a 90% reduction in new chronic HBV and HCV infections compared to the 2015 baseline”. The new guidelines by WHO use absolute targets which should be used here (World Health Organization, Interim guidance for country validation of viral hepatitis elimination, N.A.C. Geneva: World Health Organization, Editor. 2021.)**

The report provides assessment of progress towards the targets at the time. A clear reference to the new guidance that includes the new absolute targets for the impact targets has been included in the discussion.

**• Page 4, paragraph 4 – authors state “Table 2 provides a summary of the data provided by countries on the WHO European Region…”. The data in Table 2 came from other sources listed in the methodology section (WHO/UNICEF, EMCDDA report..). This should be stated clearly as these other sources have their own limitations which the reader can only understand after reading those reports.**

Text has been revised.

**• There is no mention of Table 3 in the result section.**

Text has been revised.

**• Table 3, page 17 – the data shown here is not consistent with available data in the country (national registries). For example, Sweden has a national registry that records both HBV and HCV diagnosed cases (over 80% diagnosed) but is listed as having not data. Germany has the RKI registry that has its limitations but does have reported diagnosed numbers for HCV and HBV. Same applies to Finland, Luxembourg, Italy…. Also, many of these countries (Poland, Slovakia, Luxembourg…) have national registries for the number of HCV and HBV treated patients that are not reported here.**

As noted in the methods. This is the data that has been provided to ECDC through formal routes from national contact points that are based in the ministries of health or at the public health institutes. In the case of Germany – this is the data provided to us and validated (twice) by RKI. ECDC does not have connections with local clinical registries. I have bolstered the methods section to add extra clarity around this. We are aware that there are different numbers reported from elsewhere and this is one of the challenges that need to be addressed moving forwards – to bring together all local stakeholders to together and align data sources. We have added text in the discussion to raise this important issue.  **• Page 4, paragraph 5 – authors state “estimates of the numbers with chronic hepatitis B and C were over five years old in around a third of countries..” but the authors made no effort to adjust for the impact of time. This is critical for HCV as publications in France have shown a continued decline in HCV prevalence as more people are treated. In addition, this publication should report the estimated number of HBV and HCV infections by country as this is used in the denominator of % diagnosed.**

The point made around the outdated data for HCV is a major limitation and additional detail has been added to the discussion section around this. The estimates of the numbers diagnosed are provided in full in the ECDC report that is clearly referenced.

**• Page 4, paragraph 6 – authors state “Information were obtained on the source of the data collected”. No reference for the data sources was provided.**

A reference has now been added.

**• Page 5, paragraph 6 – authors state “and of the 31 countries, 20 (65%) reported there was a plan and 10 of these countries reported that there were funds allocated from the national budget to implement the plan.” A list of countries should be provided.**

Text has been revised.  **• Page 5, paragraph 5 – “The proportion of chronic HBV and HCV cases that were diagnosed as of the end of 2017 was calculated as a fraction with the number diagnosed as the numerator and the estimated number with chronic infection as the denominator”. Again, the denominator is not shown in this manuscript.**

This paper is not intended to present all the data collected but highlight the progress towards the core indicators. Full information on all the data collected including the estimates of the numbers diagnosed are provided in full in the ECDC report that is clearly referenced.

**• Figure 6, page 24 – % diagnosed who are treated is the wrong metric that benefits countries who have a low diagnosis rate (Romania or Bulgaria). A country like France or UK treated many more people but they rank low because they have a high diagnosis rate.**

This is a good point and a clarifying sentence has been added to the discussion.

**• Figure 3, page 24 – For HCV, it is not clear if the authors are reporting a single year treated or cumulative. The WHO targets for HCV take into account cumulative HCV treated (since HCV treatment is curative). Please see the WHO report and they way the interpreted the HCV treatment rate (World Health Organization, Global progress report on HIV, viral hepatitis and sexually transmitted infections, 2021. Accountability for the global health sector strategies 2016–2021: actions for impact. 2021, World Health Organization: Geneva, Switzerland.)**

The title of the HCV treatment chart has been corrected to provided greater clarity.

The indicator used was produced according to the specifications at that time as outlined in the 2017 WHO monitoring document (referenced) in which it is clearly indicated that the treatment indicator is for one year (see below)

**• Page 15, Table 1 – it is not clear to why the goal of 90% universal coverage of HBV birth dose is not included in the core indicators for measuring progress towards the SDG targets. Every WHO guidance includes this target.**

Universal HBV birth dose is only provided in a few countries in the region as most countries have in place a universal screening programme for pregnant women and then provide selective HBV vaccination. On account of this the group of experts did not consider birth dose to be a core target.

**• Page 15, Table 1, Page 4, paragraph 3 – only programmatic targets are included. The authors state that Incidence and mortality data (impact targets) were not included because data were not available. However, on page 4, paragraph 1, the authors show Eurostat (population and mortality data) as a data source. What is this data used for? In discussion section, exclusion and plans for collection impact targets (mortality and incidence) should be discussed further as every WHO report of viral hepatitis targets cover both programmatic and impact targets.**

The EUROSTAT data were used for the calculation of other indicators that are covered in the broader ECDC report that includes a wider range of indicators. The reference to Eurostat data (and other data not presented in the paper) in the methods has now been removed. The paper provides an overview of progress towards the core indicators that were defined by an EU group. The group chose not to include the mortality and impact indicators but these are included in the broader ECDC report which is noted and referenced.
